# Longitudinal decline in semantic *versus* letter fluency, but not their ratio, marks incident Alzheimer’s disease in Latinx Spanish-speaking older individuals

**DOI:** 10.1017/S1355617722000856

**Published:** 2023-01-13

**Authors:** Kayri K. Fernández, Anton J. Kociolek, Patrick J. Lao, Yaakov Stern, Jennifer J. Manly, Jet M. J. Vonk

**Affiliations:** 1 Department of Neurology, Taub Institute for Research on Alzheimer’s Disease and the Aging Brain, Columbia University Medical Center, New York, NY, USA,; 2 Teachers College, Columbia University, Department of Biomedical Sciences, New York, NY, USA,; 3 Department of Epidemiology, Julius Center for Health Sciences and Primary Care, University Medical Center Utrecht, Utrecht University, Utrecht, The Netherlands, University of California San Francisco (UCSF), San Francisco, CA, USA; 4 Department of Neurology, Memory and Aging Center, University of California San Francisco (UCSF), San Francisco, CA, USA

**Keywords:** category fluency, phonemic fluency, language, dementia, Hispanic

## Abstract

**Objective:**

To compare longitudinal verbal fluency performance among Latinx Spanish speakers who develop Alzheimer’s disease to those who do not develop dementia in absolute number of words produced on each task and their ratio to combine both scores.

**Method:**

Participants included 833 Latinx Spanish-speaking older adults from a community-based prospective cohort in Manhattan. We performed growth curve modeling to investigate the trajectories of letter and semantic fluency, and their ratio (i.e., ‘semantic index’), between individuals who developed Alzheimer’s disease and those who did not (i.e., controls). The semantic index quantifies the proportion of words generated for semantic fluency in relation to the total verbal fluency performance.

**Results:**

Letter fluency performance did not decline in controls; we observed a linear decline in those who developed Alzheimer’s disease. Semantic fluency declined in both groups and showed an increased rate of change over time in the incident Alzheimer’s disease group; in comparison, the control group had a linear and slower decline. There were no group differences in the longitudinal trajectory (intercept and slope) of the semantic index.

**Conclusion:**

A decline in letter fluency and a more rapid and accelerating decline over time in semantic fluency distinguished people who developed Alzheimer’s disease from controls. Using the semantic index was not a superior marker of incident Alzheimer’s disease compared to examining the two fluency scores individually. Results suggest the differential decline in verbal fluency tasks, when evaluated appropriately, may be useful for early identification of Alzheimer’s disease in Latinx Spanish speakers, a historically understudied population.

## Introduction

Verbal fluency tests are common measures of cognitive function that are sensitive to decline with age and several neurodegenerative diseases, including different types of dementia (Clark et al., 2009; Shao et al., 2014). Despite the popularity of these tests, research has historically concentrated on convenience or clinical samples that were often small in size with homogeneous socioeconomic and cultural characteristics (Lara et al., 2021). Epidemiological studies suggest substantial differences in prevalence and incidence rates of Alzheimer’s disease among ethnoracial groups (Chin et al., 2011), including a higher incidence rate for Latinx individuals compared to White non-Hispanic individuals (Tang et al., 2001). Understanding cognitive test performance among individuals who are not non-Latinx White and English-speaking is thus imperative to improve diagnostic accuracy of measures for neurodegenerative disorders such as Alzheimer’s disease.

Verbal fluency tasks require generation of as many different words within a given time (e.g., 1 min) for a given criterion. Typically, this criterion is that words start with a particular letter (e.g., C, F, or L in English-speaking individuals), called “letter fluency”, or belong to a particular category (e.g., animals), called category or “semantic fluency” (Eng et al., 2019). While both measures tap into lexical retrieval, speed, and executive function, semantic fluency relies more heavily on semantic memory (i.e., knowledge of facts and concepts, including the meaning of words), while letter fluency relies more heavily on executive functioning (Rascovsky et al., 2007; Shao et al., 2014; Vonk et al., 2019).

Cognitively normal individuals typically produce more words in semantic than letter fluency, while individuals with Alzheimer’s disease often perform worse, relative to cognitively normal performance, on semantic than letter fluency (Crossley et al., 1997; Rascovsky et al., 2007; Salvatierra et al., 2007). Because of this reversal in performance pattern between the tasks, the differential performance on semantic *versus* letter fluency is often used in the clinical diagnosis of Alzheimer’s disease (Clark et al., 2009; Malek-Ahmadi et al., 2011). One downside of this approach is that the relative difference between the two tasks is hard to quantify. Therefore, Rascovsky et al. (2007) developed the ‘semantic index’, combining both tasks into one metric, to illustrate this difference in output between letter and semantic category fluency to aid in the differential diagnosis between Alzheimer’s disease and frontotemporal dementia. The semantic index is a formula that quantifies the proportion of words generated for semantic fluency in relation to the overall verbal fluency performance on both task conditions: $\text{semantic}\;\text{index}\;=\frac{semantic\;fluency}{\left(semantic\;fluency\;+\;letter\;fluency\right)}$. They showed that individuals with autopsy-confirmed Alzheimer’s disease and frontotemporal dementia exhibited disparate verbal fluency performance at similar stages of illness: individuals with frontotemporal dementia had a higher semantic index (they produced more words on semantic than letter fluency) than those with Alzheimer’s disease (who produced more words on letter than semantic fluency). This result was replicated in a study by Yoshizawa et al. (2013).

The semantic index measure may have potential to capture differential relative semantic *versus* letter fluency performance into one metric beyond the manifest dementia groups in which it has been tested. Specifically, it would be of interest to investigate if the semantic index could capture the flip in verbal fluency performance across the continuum from healthy aging to dementia, including the preclinical stage of Alzheimer’s disease, as this longitudinal change is a function of time and disease progression. Moreover, sociodemographic influences—including ethnicity, sex/gender, and education—on this metric are not yet well-understood. Neuropsychological measures are known to lack diagnostic accuracy when used in individuals from historically excluded populations (American Psychiatric Association & DSM-III Work Group to Revise, 1987; American Psychological Association, 2002; Ardila et al., 2002; Harris, 2002; Manly, 2008). To identify when cognitive performance deviates from normal aging towards the earliest signs of dementia among individuals from all backgrounds, we need more data on the longitudinal neurospychological performance of individuals from cultural and linguistic backgrounds other than a non-Latinx White English-speaking background.

The current study investigated the trajectory of letter fluency, semantic fluency, and their ratio as calculated by the semantic index in Latinx Spanish-speakers between two groups: individuals who developed incident Alzheimer’s disease and those who did not develop dementia during follow-up. We aimed to describe how letter and semantic fluency independently function over time, and if the semantic index can capture their flip in performance over time into one metric in preclinical Alzheimer’s disease. We investigated these patterns in a long-running community-based sample of Latinx Spanish-speaking individuals, who have historically been understudied.

## Methods

### Participants

Participants were drawn from the Washington Heights-Inwood Columbia Aging Project (WHICAP) in northern Manhattan. WHICAP is a multi-ethnic community-based prospective cohort study designed to observe epidemiological changes in cognitive aging and dementia. Recruitment occurred across three waves (i.e., 1992, 1999, 2009) (Kulick et al., 2020; Zahodne et al., 2019). In 1992, the first wave of participants was recruited via random sampling of Medicare-eligible older adults residing in selected census tracts of the Washington/Hamilton Heights and Inwood neighborhoods in New York City. Participants were eligible to participate if they were aged 65 years or older, fluent in Spanish or English, willing to be visited for an in-person interview and examination, and willing to be followed longitudinally. The second (1999) and third (2009) recruitment waves were recruited from the same communities with the same eligibility criteria, except that participants were excluded if they reported a dementia diagnosis or had serious memory complaints at screening. Moreover, recruitment in the second and third wave was aimed to produce a final cohort that would be equally divided among Latinx, non-Latinx Black, and non-Latinx White participants, and represent equal proportions of 65–74 and ≥75-year-old participants at enrollment.

At time of enrollment, participants were asked about their predominant language spoken at home, their first language spoken (i.e., native language), and their English proficiency. Subsequently, the interviewer asked the following question to determine in which language to perform the battery of tests: “Now I want to know whether you think you can perform your best on these tests in English or Spanish. This is the language you are most comfortable, fluent, and familiar speaking. Keep in mind that we will do the entire interview in the language that you choose, Spanish or English.” After participants chose their preferred language, the entire battery of tests was performed in that language, including follow-up visits.

Participants were evaluated every 1.5–2 years using a series of physical, neurological, and neuropsychological exams to elucidate risk factors associated with dementia (Stern et al., 1992; Tang et al., 2001). All participants provided written consent following the Institutional Review Boards of Columbia University Medical Center and Columbia University Health Sciences and the New York State Psychiatric Institute. Research for this present study was conducted and completed in accordance with the Helsinki Declaration.

For the current study, participants were selected if they were Latinx and assessed in Spanish. Most participants emigrated to northern Manhattan and were born in one of the following Latin American countries or regions: Central America (*n* = 31), Cuba (*n* = 121), Dominican Republic (*n* = 521), Mexico (*n* = 3), Puerto Rico (*n* = 101), and South America (*n* = 45). The rest of the Spanish-speaking participants was born in parts of Europe (n = 4), the United States (*n* = 3), or did not report birthplace (*n* = 4). All 833 selected participants identified as Latinx; race/ethnicity was self-reported following the 1990–2000 US census format (United States Bureau of the Census, 1991).

**Figure 1.**
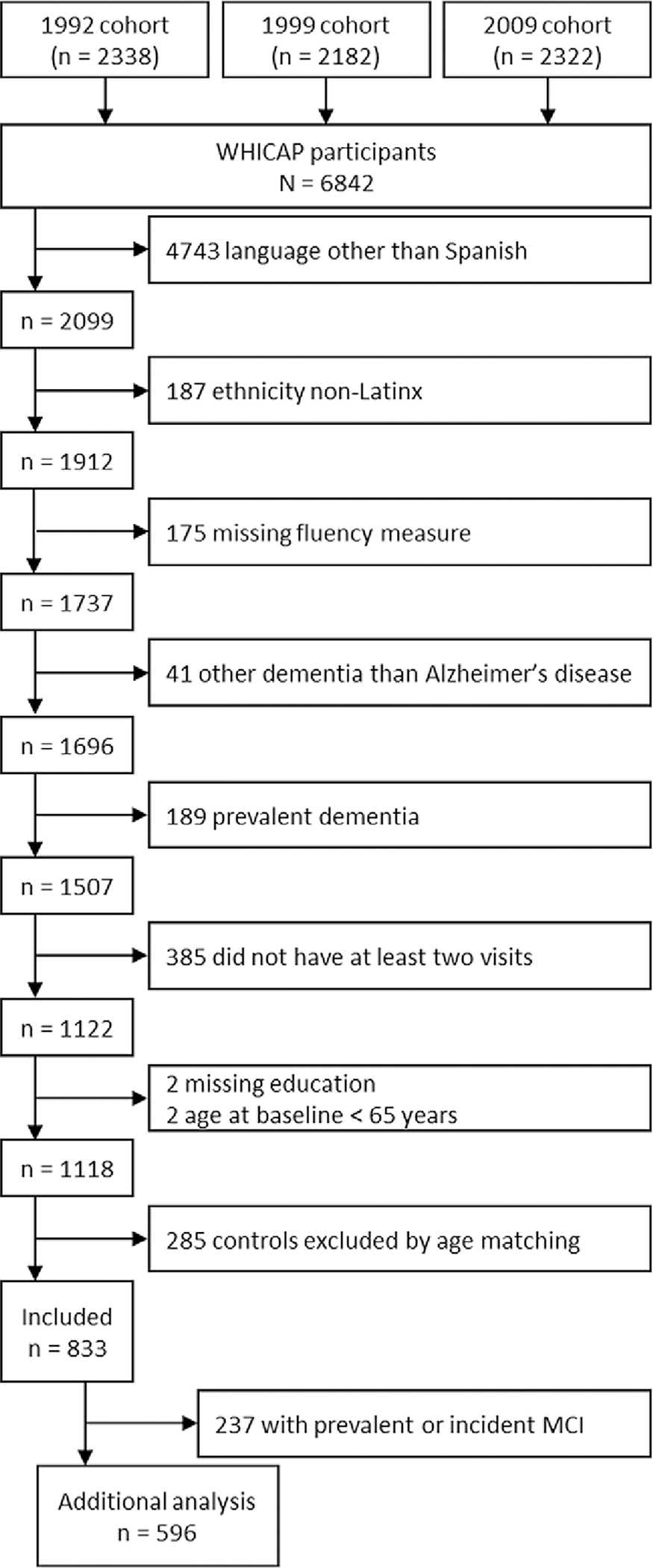
Flowchart participant selection.

Participants were included if they were assessed in Spanish, Latinx, age 65 or older, had no missing data for both verbal fluency measures at baseline, had no missing data for years of education, had a minimum of two visits, and had a known diagnostic status of either no dementia throughout follow-up or incident Alzheimer’s disease. Exclusion criteria were prevalent dementia and incident dementia that was not clinically diagnosed as Alzheimer’s disease. Incident dementia diagnosis was classified as the first-time participants in the study went from cognitively normal or having mild cognitive impairment to a diagnosis of Alzheimer’s disease; this criterion thus led us to exclude individuals with only one visit. Because the available sample of controls was on average younger than the incident Alzheimer’s disease group, we performed a matching procedure to match the diagnostic groups on age at baseline. The final sample included 300 older individuals with an incident Alzheimer’s disease diagnosis and 533 participants who remained without a dementia diagnosis during follow-up (Figure 1).

## Materials and procedures

### Determinant

The independent variable of interest (i.e., determinant) was incident Alzheimer’s disease diagnosis. Dementia diagnosis, including Alzheimer’s disease and other types, was established at each visit by an interdisciplinary team of neuropsychologists and neurologists following standard criteria (American Psychiatric Association & DSM-III Work Group to Revise, 1987; McKhann et al., 1984).

### Outcome variables

Participants completed letter and semantic fluency tasks at each study visit as part of a neuropsychological battery of tests, described in detail elsewhere (Jacobs et al., 1997; Siedlecki et al., 2008). For letter fluency, participants were asked to generate as many words as possible in 1 min that started with a specific letter—namely P, S, or V—across three separate trials. The rationale of using the letters P, S, and V for the Spanish version of this task is detailed in Jacobs et al. (1997) and these letters are commonly adopted to perform this task in Spanish (e.g., Weir et al., 2014). The mean raw score across these three trials represented the score for letter fluency. For semantic fluency, participants were asked to generate as many items as possible in 1 min for the category animals; the raw score represented the score for semantic fluency. Participants were instructed that no credit would be given for the same word with a different ending (e.g., if they say “run”, they cannot also say “running”, “runs”, “ran”, or if they say “cat” they cannot also say “cats”), for proper names (e.g., “Robert” or “Rome”). If code-switching occurred (e.g., participant started saying words in English), the interviewer did not correct the participant and the word was counted as incorrect when scored. The letter and semantic fluency instructions were translated into Spanish by qualified native Spanish speakers from Cuba, Puerto Rico, Spain, and the Dominican Republic (Jacobs et al., 1997).

For each individual, we calculated the semantic index developed by Rascovsky and colleagues, as described earlier (Rascovsky et al., 2007). The semantic index formula quantifies the proportion of words generated for semantic fluency in relation to overall verbal fluency performance on both task conditions. For example, a semantic index of 0.5 indicates that an equal number of words was generated for semantic and letter fluency, an index over 0.5 indicates that more words were generated for semantic than letter fluency, and an index below 0.5 indicates that more words were generated for letter than semantic fluency. For modeling purposes, the index was multiplied by 100 to represent a percentage.

### Covariates

Age at baseline visit was calculated as the date at first visit minus date of birth and was used with one decimal place accuracy. Recruitment wave (i.e., 1992, 1999, 2009) was coded as a three-level categorical variable, as the recruitment process slightly varied across waves (see “Participants”). Sex/gender and educational attainment were recorded based on self-report. Sex/gender was recorded as a dichotomized variable (men vs. women); we refer to this variable as “sex/gender” because we could not establish whether participants reported their gender identity or their biological sex when asked (Avila et al., 2019). Educational attainment was reported in years and was used continuously.

### Statistical analysis

Sample characteristics were calculated using descriptive statistics, general linear models, and chi-square tests in R version 4.1.1 (R Core Team, 2021) using the “furniture” package (Barrett & Brignone, 2017). We performed univariate latent growth curve modeling in Mplus version 8 (Muthén & Muthén, 2019) to investigate the trajectories of verbal fluency performance between Spanish-speaking individuals who developed incident Alzheimer’s disease over time and those who did not develop dementia (i.e., controls). Latent growth curve models are a statistical technique that can be used on longitudinal data to study between-person differences in within-person change (Curran et al., 2010). A person’s trajectory may increase, decrease, or remain flat over time, and may be linear or curvilinear (i.e., having an acceleration or deceleration of change throughout the trajectory). A latent growth curve model estimates the mean trajectory across a defined set of individuals that is composed of the mean intercept (i.e., typically level at first measurement, but in this study intercept represents level at last measurement) and mean slope (i.e., change over time).

We performed multiple-group growth curve models with diagnosis (incident Alzheimer’s disease *vs*. control) as the grouping variable. Multiple-group analysis allows parameters, including variances and covariances, to be estimated independently across different groups. Change in verbal fluency score (semantic fluency, letter fluency, or the semantic index) across up to six visits was used as the outcome. Models applied a maximum likelihood estimation with standard error approximation using the first-order derivative, and were estimated with random intercepts and slopes for time. With the diagnostic groups matched on age at baseline, we modeled time as years before diagnosis for those who develop incident Alzheimer’s disease or years to last follow-up for those who did not (Weuve et al., 2015). We incorporated individually-varying time scores to account for varying intervals between visits; the scale of the time variable is in years with one decimal place accuracy. The covariates were centered to obtain parameter estimates for the verbal fluency measures reflecting the sample’s mean intercept and slope. Differences in intercept and slope between diagnostic groups was tested with Wald z tests through model constraints. The significance level for all tests performed in this study was set *a priori* at α = .05. The reported coefficients reflect unstandardized effects.

We started with an unconditional latent growth curve models to compare model fit of linear *versus* quadratic change over time. Modeled for each diagnostic group separately, lower BIC values between nested models indicated that linear models provided a better fit than those including a quadratic effect for letter fluency (control linear BIC 8737.512 *vs*. quadratic BIC 8761.022; Alzheimer’s disease linear BIC 5588.716 *vs*. quadratic BIC 5601.274) and the semantic index (control linear BIC 14253.049 *vs*. quadratic BIC 14277.081; Alzheimer’s disease linear BIC 9607.604 *vs*. quadratic BIC 9623.860). For semantic fluency, a linear model was a better fit for the control group (linear BIC 14253.049 *vs*. quadratic BIC 14277.081), but a quadratic model was a better fit for the incident Alzheimer’s disease group (linear BIC 9607.604 *vs*. quadratic BIC 9623.860). A nonlinear decline consists of both a linear slope and a quadratic slope, in which the linear slope represents the instantaneous change per time unit (i.e., change at the intercept) and the quadratic slope represents the acceleration of the change. Both elements together represent the non-linear change, however, the effects of diagnostic group on the change over time are statistically tested on each element separately.

**Figure 2.**
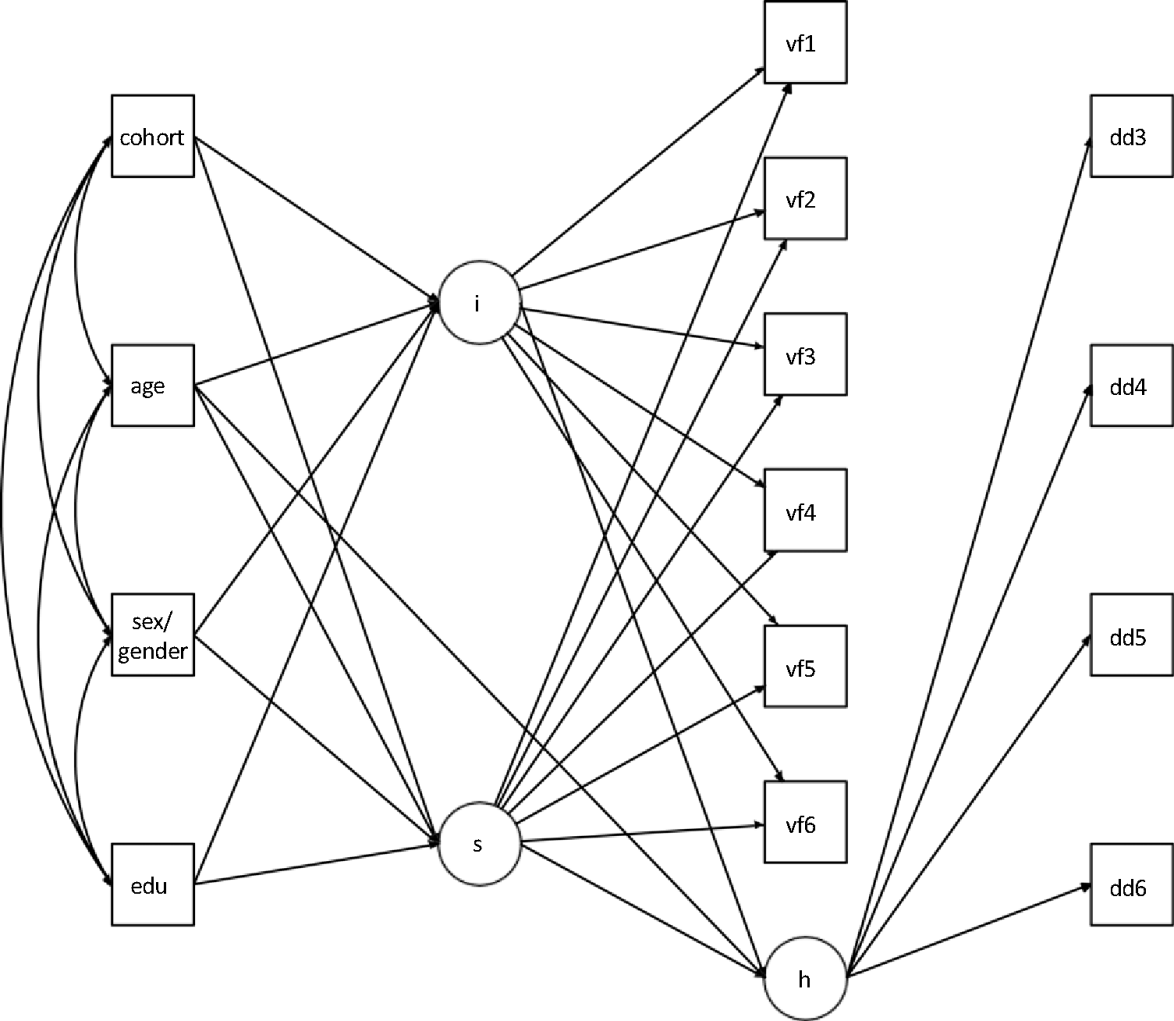
Measurement model (i = intercept; s = slope; vf = verbal fluency; h = latent hazard function; dd = death and drop-out).

Subsequently, we performed conditional multiple-group models for each outcome (i.e., letter fluency, semantic fluency, or the semantic index) with age at baseline, recruitment wave, sex/gender, and education as covariates. To account for bias due to drop-out and death (i.e., informative censoring), we jointly modeled the conditional probability of death or drop-out at a specific visit, given survival and no drop-out at previous visits, as a discrete-time survival model within the growth model. The latent hazard function was adjusted for age at baseline and regressed on the latent growth parameters (intercept and slope) to adjust the trajectory estimates for informative censoring. We performed an additional analysis in which we excluded all individuals with a diagnosis of mild cognitive impairment (MCI) at any time during study participation from the control group. The general measurement model, including covariates and hazard function, is depicted in Figure 2. All Mplus code and output is available on GitHub: https://github.com/jmjvonk.

## Results

### Sample characteristics

In the total sample (*N* = 833), participants were on average 78.0 (*SD* = 5.2) years old, 70.8% were women, and the average years of education was 6.6 (*SD* = 4.0) (Table 1). At their first visit, participants produced on average 11.7 (*SD* = 3.8) words on semantic fluency and 7.0 (*SD* = 3.2) words on letter fluency. The number of visits was on average 3.8 (*SD* = 1.4) across an average follow-up time of 6.7 years (*SD* = 3.7). Retention rates were 100% at visit 2 (*n* = 833), 74.5% at visit 3 (*n* = 621), 74.2% at visit 4 (*n* = 461), 57.5% at visit 5 (*n* = 265), and 56.2% at visit 6 (*n* = 149). Table 1 present participant characteristics between diagnostic groups.

### Trajectories of verbal fluency performance

**Figure 3.**
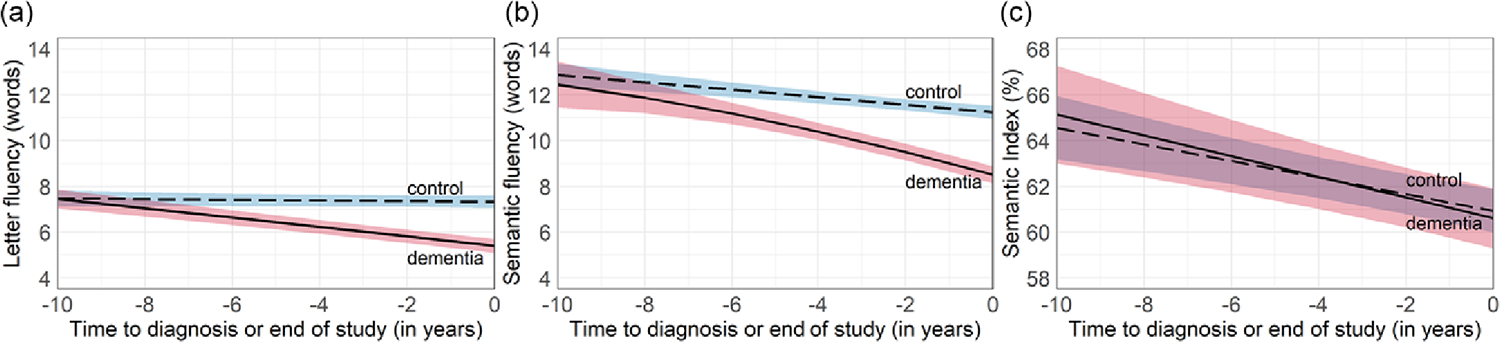
Trajectories of (a) letter fluency, (b) semantic fluency, and (c) semantic index across diagnostic groups.

Parameter estimates for all three models are provided in Table 2, and trajectories between diagnostic groups are shown in Figure 3. For letter fluency, the conditional (fully adjusted) model showed a higher mean level of performance at the end of the study (i.e., intercept) for the control group than the incident Alzheimer’s disease group (ΔB = 1.919, *SE* = .205, *p* < .001). The control group did not show decline in their letter fluency performance over time, while the incident Alzheimer’s disease group did (ΔB = .190, *SE* = .025, *p* < .001); the incident Alzheimer’s disease group declined on average .205 words per year, which translated to on average nearly half a word less per visit if a participant was evaluated every 2 years in WHICAP, i.e., one word less per 5 years.

For semantic fluency, the conditional model also showed a higher mean level of performance at the end of the study (i.e., intercept) for the control group than the incident Alzheimer’s disease group (ΔB = 2.725, *SE* = .238, *p* < .001). The linear slope of the control group showed a decline, but this decline was less steep than in the incident Alzheimer’s disease group (ΔB = .359, *SE* = .047, *p* < .001). The control group declined on average .164 words per year, which translated to on average one word less per 6 years. Meanwhile, the incident Alzheimer’s disease group declined on average .523 words per year, which translated to on average more than one word less per visit if a participant was evaluated every 2 years in WHICAP. Moreover, we observed an acceleration in decline in the incident Alzheimer’s disease group beyond what was predicted by the linear factor (i.e., quadratic slope), i.e., faster decline closer to diagnosis compared to earlier years. We did not observe this acceleration in decline in the control group towards their last observed visit.

The conditional model for the semantic index showed no difference in intercepts between diagnostic groups (ΔB = .324, *SE* = .826, *p* = .695), which unit represents the percentage of words generated for semantic fluency in relation to overall verbal fluency (letter + semantic) performance. Both groups showed a similar linear decline in semantic index (ΔB = .090, *SE* = .118, *p* = .447).

The additional analysis with models that only included individuals in the control group that did not receive a diagnosis of MCI at any visit during their follow-up suggests that this subgroup of controls performed slightly better on letter and semantic fluency in the years before the end of study than the controls with MCI diagnosis. Nonetheless, the overall patterns in all three models are similar to the original model, including no difference across groups in the semantic index measure.

## Discussion

Understanding how cognitive measures perform in people who are usually excluded from research on cognitive function in older adults is imperative for the accurate and timely diagnosis of Alzheimer’s disease for individuals from all backgrounds. This study aimed to describe the longitudinal trajectories of letter and semantic fluency as well as their ratio, captured by the semantic index (Rascovsky et al., 2007), in Latinx Spanish-speaking individuals of whom a subset developed incident Alzheimer’s disease. Our results show a differential decline on letter *versus* semantic fluency between people who do not develop dementia and people who develop Alzheimer’s disease. Inclusion of the semantic index in our analyses was specifically intended to highlight differential performance on semantic *versus* letter fluency between the two diagnostic groups by combining both tasks into one metric. In contrast to our expectations, however, the results showed that the semantic index did not capture this differential verbal fluency performance in semantic fluency *versus* letter fluency in the earliest stages of Alzheimer’s disease. A more sensitive tool is needed to capture and quantify this dissociation between tasks over time.

While a (non-linear) pattern of decline in semantic fluency for the incident Alzheimer’s disease group is not surprising (Vonk et al., 2020), our control group also showed linear decline on semantic fluency, even when restricted to individuals without MCI diagnosis. The extent of decline in letter and semantic fluency trajectories in older adults without dementia remains disputed (Holtzer et al., 2020; Kave & Knafo-Noam, 2015; Vonk et al., 2020). Future studies on modeling the trajectory of letter and semantic fluency are warranted—particularly in samples with a diverse representation in terms of race/ethnicity and education—because establishing the pattern in healthy aging will aid development of measures to determine when a deviation from the expected pattern occurs that marks dementia.

In our study, calculation of the semantic index (i.e., ratio) contributed to a loss of information about fluency performance in the preclinical dementia stage, while previous studies in clinical dementia samples showed this index to be an effective measure for differentiating individuals with frontotemporal dementia from those with Alzheimer’s disease (Rascovsky et al., 2007; Yoshizawa et al., 2013). The main clinical differences in the samples between the studies are that we aspired to generalize the semantic index to earlier stages of Alzheimer’s disease (i.e., preclinical), and that we compared individuals with Alzheimer’s disease to cognitively normal individuals instead of another type of dementia. Therefore, using a ratio in our sample may be less appropriate for two reasons. Firstly, by expressing the performance on semantic fluency as a proportion of total fluency (semantic + letter fluency), there is an implicit assumption that semantic fluency changes while letter fluency performance stays relatively stable. We showed that letter fluency performance remained stable over time in the control group but declined in the group with incident Alzheimer’s disease. Secondly, the ratio between letter and semantic fluency does not take a non-linear decline in semantic fluency into account, which complicates interpretation of the ratio’s value at different timepoints in the disease process if we investigate the ratio over time. Besides these differences, the tasks also rely on common processes including lexical retrieval, speed, and executive function that may lead to effortful retrieval in the participants with Alzheimer’s disease relative to controls across both tasks (Shao et al., 2014).

A third observation was that the semantic index remained on average above 0.5 (or above 50%) throughout follow-up in both groups, including the visit at which a diagnosis was established in those who developed Alzheimer’s disease. Meanwhile, the mean semantic index for individuals with manifest Alzheimer’s disease was below 0.5 in both Rascovsky et al. (2007) and Yoshizawa et al. (2013). A value of over 0.5 or 50% means that even in participants who developed incident Alzheimer’s disease, the number of words was higher on semantic fluency than letter fluency at their diagnostic visit. However, in accordance with both Rascovsky et al. (2007) and Yoshizawa et al. (2013), we found that the Alzheimer’s disease group generated fewer words for semantic fluency in comparison to the controls. Our study differed from the previous two studies by using a longitudinal approach during a time frame years before diagnosis, while Rascovsky et al. (2007) and Yoshizawa et al. (2013) cross-sectionally studied individuals who had been diagnosed with Alzheimer’s disease years prior to testing. Using this longitudinal approach in preclinical stages, our results further highlight the development of the flip in pattern over time, including the non-linearity of the decline in semantic fluency performance.

Strengths of this study include a large sample size of Latinx Spanish-speaking individuals, which allowed us to perform a longitudinal analysis across a relatively long follow-up period in a historically understudied population. Additionally, the range of years of education was wider in our sample than in the majority of other cohort studies, including a large group of individuals with fewer than 9 years of school. The wider range in education is a result of the community-based recruitment approach of WHICAP, which allows for better generalizability of results than clinical or convenience sampling approaches. In addition to years of education, future studies may also want to investigate the effect of quality of education on verbal fluency performance over time.

A limitation of this study is that our participants were recruited from northern Manhattan, a primarily Caribbean-descent neighborhood of mostly Dominican and Puerto Rican immigrants, therefore limiting the generalizability to urban and American-born Latinx individuals or individuals from other Latin American countries. The immense cultural heterogeneity in Latin American countries and the Caribbean means that Hispanic/Latinx individuals should never be considered a uniform group. Another limitation includes our approach to model reverse time (i.e., time in years before diagnosis). This approach allowed us to focus on the preclinical phase of Alzheimer’s disease by knowing their future diagnosis. However, by definition using reverse time requires selection criteria in terms of follow-up time and disease outcome, which may introduce bias in the parameter estimates (Weuve et al., 2015). Additionally, by creating groups based on diagnosis, individuals may have been included in the control group whose eventual Alzheimer’s disease diagnosis was not captured due to death or loss-to-follow in WHICAP (e.g., moving to higher level of care and not being able to participate anymore), or was not yet captured as they may convert in the future. These possible scenarios may have contributed to the observed group differences in the number of visits and length of time in the study. We recognize that verbal fluency tests are used to determine diagnosis, introducing a degree of circularity in the analyses: by definition, because their cognitive scores fell below dementia cut-off scores at a subsequent follow-up visit, the incident Alzheimer’s disease group at diagnosis most likely had lower fluency scores than: (1) themselves at baseline; and (2) the control group at last visit. Lastly, while verbal fluency tests are free of ceiling effects, they may be subject to floor effects. In our data, participants who developed dementia were producing few words on the letter fluency task by the end of the study, which may have complicated correct computation of the shape of decline. Nonetheless, the pattern of a linear decline in letter fluency *versus* a curvilinear decline in semantic fluency in individuals with Alzheimer’s disease matches the pattern we found in our previous work in a different cohort (Vonk et al., 2020).

This study showed that a decline in letter fluency and a more rapid decline in semantic fluency distinguished people who developed Alzheimer’s disease from controls, in line with previous literature (e.g., Holtzer et al., 2020). In contrast to our expectations, we were not able to capture this dissociation and change between task performance over time by combining letter and semantic fluency into one metric using the semantic index in the preclinical stage of Alzheimer’s disease. Instead, we found that examination of the two fluency scores individually, in Spanish-speaking Latinx older adults, provided more insight into verbal fluency decline over time in participants who developed Alzheimer’s disease *versus* those who did not develop dementia. Revealing this difference between verbal fluency tasks in a historically understudied population contributes to finding appropriate measures for early identification of individuals who will ultimately be diagnosed with Alzheimer’s disease in diverse populations.

## Figures and Tables

**Table 1.** Participant characteristics across diagnosis groups at baseline

	Control *n* = 533	Incident Alzheimer's disease *n* = 300	*p*-value

Age	77.9 (4.8, 65.6–92.7)	78.2 (5.8, 65.2–94.4)	.421
Sex/gender: women	366 (68.7%)	224 (74.7%)	.080
Education	7.0 (4.0, 0–20)	5.9 (3.9, 0–20)	<.001
Time in study	6.3 (3.7, 0.9–22.6)	7.3 (3.8, 0.9–23.1)	<.001
Number of visits	3.6 (l.4, 2–6)	4.1 (1.4, 2–6)	<.001
Semantic fluency	12.2 (3.7, 2–25)	10.9 (3.8, 2–26)	<.001
Letter fluency	7.3 (3.2, 0.7-l8.3)	6.5 (3.2, 0.3–26.7)	<.001
Semantic index	63.0 (l0.6, 31.6–93.1)	63 (12.9, 18.8–96.8)	.950

*Note.* Measures are mean (standard deviation; range) for continuous variables and number (%) for categorical variables.

**Table 2.** Mean estimates for intercept and slope, and estimates for the effect of diagnostic group on letter fluency, semantic fluency, and the semantic index

		Unconditional model		Conditional model		Additional analysis (no MCI)	
		Control (*n* = 533)	Alzheimer's disease (*n* = 300)	Control (*n* = 533)	Alzheimer's disease (*n* = 300)	Control (*n* = 296)	Alzheimer's disease (*n* = 300)

Letter	Intercept	7.288 [7.001, 7.576]*	5.359 [5.055, 5.663]*	7.324 [7.061, 7.587]*	5.406 [5.099, 5.713]*	8.045 [7.651, 8.439]*	5.405 [5.099, 5.712]*
	Linear slope	−.037 [−.066, −.008]*	−.235 [−.281, −.189]*	−.015 [−.048, .018]	−.205 [−.243, −.167]*	−.009 [−.058, .040]	−.202 [−.241, −.164]*
Semantic	Intercept	11.202 [10.911, 11.493]*	8.443 [8.083, 8.804]*	11.237 [10.947, 11.526]*	8.512 [8.146, 8.877]*	11.802 [11.418, 12.185]*	8.512 [8.146, 8.878]*
	Linear slope	−.181 [−.222, −.139]*	−.595 [−.697, −.493]*	−.164 [−.221, −.107]*	−.523 [−.593, −.454]*	−.190 [−.266, −.115]*	−.522 [−.593, −.451]*
	Quadratic slope	−	−.022 [−.033, −.012]*	−	−.013 [−.025, −.001]*	−	−.013 [−.025, .000]*
Semantic index	Intercept	61.210 [60.253, 62.166]*	60.511 [58.985, 62.037]*	60.934 [59.991, 61.877]*	60.610 [59.294, 61.926]*	59.798 [58.412, 61.183]*	60.610 [59.293, 61.927]*
	Linear slope	−.207 [−.321, −.093]*	−.320 [−.508, −.131]*	−.363 [−.507, −.219]*	−.453 [−.646, −.260]*	−.425 [−.603, −.247]*	−.455 [−.647, −.262]*

*Note*. Conditional Model = adjusted for age at first visit, recruitment cohort, sex/gender, and education; the additional analysis which excludes individuals with MCI is performed on the conditional model.

* *p* < .05.
